# Efficiency of biological versus physical optimization for single‐arc VMAT for prostate and head and neck cases

**DOI:** 10.1120/jacmp.v15i4.4514

**Published:** 2014-07-08

**Authors:** Vadzim Pyshniak, Irina Fotina, Alena Zverava, Stanislau Siamkouski, Elena Zayats, Georgy Kopanitsa, Dzmitry Okuntsau

**Affiliations:** ^1^ Gomel Regional Oncology Center Gomel Republic of Belarus; ^2^ Institute of Physics and Technology Tomsk Polytechnic University Tomsk Russia; ^3^ Elekta GmbH Innsbruck Austria; ^4^ Institute Cybernetic Center Tomsk Polytechnic University Tomsk Russia; ^5^ Tomsk State University for Architecture and Building Tomsk Russia

**Keywords:** VMAT, IMRT, biologically based optimization, dose‐volume‐based optimization, plan comparison, prostate cancer, head and neck cancer

## Abstract

The aim of this work was to compare different approaches to VMAT optimization (biological vs. physical DVH‐based) in two commercial treatment planning systems (TPS) for head and neck and prostate cases, using Pareto fronts. VMAT vs. IMRT Pareto front comparison was additionally performed in order to benchmark the optimizer efficiency and VMAT plan quality for each TPS. Three prostate and three head and neck cancer patients were selected for nine‐beam IMRT and single‐arc VMAT planning in Monaco 3.00 and Oncentra MasterPlan (OMP) 3.3 planning systems. Pareto fronts for prostate cases were constructed based on PTV coverage by 95% isodose and volume of rectum receiving 60 Gy or more. For head and neck cases, PTV coverage by the same isodose and mean dose to parotid gland were used for the construction of Pareto fronts. DVH analysis was performed together with evaluation of planning and delivery efficiency for all the plans. In the intersystem comparison for prostate plans, Monaco generated very similar IMRT and VMAT solutions. Quality of Monaco VMAT plans was superior compared to Oncentra in terms of conformity, homogeneity, and lower median dose to bladder due to biological formalism of optimization cost functions. For the head and neck cases, IMRT and VMAT plans were similar in both systems, except the case where a very strong modulation was required. In this situation single‐arc VMAT plan generated with OMP was inferior compared to IMRT. VMAT OMP solutions were similar to Monaco or slightly better for two less‐modulated head and neck cases. However, this advantage was achieved on the cost of lower conformity and homogeneity of the Oncentra VMAT plans. IMRT and VMAT solutions generated by Monaco were very similar for both prostate and head and neck cases. Oncentra system shows a bigger difference, and use of the dual‐arc VMAT would be recommended to achieve the same plan quality as nine‐field IMRT. Biological optimization seems beneficial in terms of plan conformity and homogeneity and allowed achieving lower OAR doses for prostate cases. In complex anatomical situations represented by head and neck cases, sequencing algorithm in Monaco imposed limitations on VMAT plan quality in the intersystem comparison.

PACS numbers: 87.55.de, 87.55.dk, 87.53.Jw

## I. INTRODUCTION

Intensity‐modulated radiotherapy (IMRT) with inverse plan optimization allows designing treatment plans with delivery of the high dose to the target volumes and steep dose gradients, while sparing critical structures. Volumetric‐modulated arc therapy (VMAT) promises further advantages compared to fixed‐gantry IMRT techniques due to simultaneous variation of the gantry speed, dose rate, and multileaf collimator (MLC) segments.^1^, ^2^ Popularity of VMAT increased dramatically after appearance and availability of the number of commercial solutions for VMAT planning and their delivery on the radiotherapy market.^3^, ^4^, ^5^, ^6^

Various commercial treatment planning systems feature different approaches to the VMAT optimization and sequencing, adding a certain degree of complexity in the VMAT planning process. Whereas the advantage of VMAT in terms of speed of treatment delivery is already well‐known to users, the question about the quality of VMAT plans is still open and strongly depends on the approach to plan optimization and sequencing. Several planning studies were performed for the comparison of fixed‐gantry IMRT versus VMAT for different clinical sites. ^7^, ^8^, ^9^, ^10^, ^11^, ^12^ Depending on the complexity of the cases and anatomical region, as well as on the applied treatment planning system, the quality of the VMAT plans ranged from inferior through equal to superior, compared to IMRT plans.

However, there is evidence that application of equivalent uniform dose (EUD) formalism of the cost functions for the IMRT optimization can improve the plan quality and offer the possibility to establish class solutions for the group of cases.^13^, ^14^, ^15^, ^16^ Recently, efficiency of the biological formalism for VMAT was assessed by Mihaylov et al.^(17)^ On the other hand, these comparisons can't provide the definitive answer about superiority of VMAT plans in terms of quality because usually one IMRT (VMAT) plan is created per patient case. In this scenario, there exists no guarantee that “the best possible” solution was found by the optimizer for each case.

The idea of using Pareto fronts as a tool for comparison in radiotherapy planning studies was tested by research groups of Ottosson et al.^18^, ^19^ and Petersson et al.,^(20)^ and this evaluation concept provides a possibility to compare a set of plans minimizing such effects as planner dependence or specific plan selection. Sampling the Pareto fronts by variation of single parameter — for example, maximum dose to the risk organ while keeping other parameters constant — will provide the information about optimizer efficiency in terms of fulfilling this constraint. Therefore it would be possible to evaluate and define more advantageous optimization approaches, together with preferable delivery technique, for a group of cases.

In the situation where resources for the treatment planning are limited (either in terms of number of workstations or time available for planning), it is important to select a planning system that provides good optimization results for the variety of clinical situations. Plan quality and delivery efficiency for VMAT, together with the time required to create a desired plan, become important criteria for the choice of the TPS for VMAT implementation as a routine technique to substitute IMRT. The aim of this work was to compare different approaches to VMAT optimization (biological vs. physical DVH‐based) in two commercial treatment planning systems for head and neck and prostate cases, using Pareto fronts. VMAT versus IMRT Pareto front comparison was additionally performed in order to benchmark the optimizer efficiency and VMAT plan quality for each TPS. Based on the obtained results, planning workflow in our institution was organized in a more efficient way, distributing the cases according to the anatomical group and plan intent for IMRT and VMAT cases between both systems.

## II. MATERIALS AND METHODS

### A. Patient cases

Three prostate and three head and neck (H&N) cases were selected for the retrospective treatment planning study from the clinical database. CT images were acquired with a multislice CT scanner (Siemens Healthcare, Erlangen, Germany) in a spiral mode with 2 mm slice thickness for H&N and 4 mm for prostate cases. Contouring of the cases was done in Monaco TPS (Elekta Corporation, Atlanta, GA) to avoid differences in the systems with regard to the contour interpolation.

The cases with prostate carcinoma represented typical indication for definitive radiotherapy (low‐risk, T1‐T2a, Gleason score ≤6 and PSA <10). The PTV encompassed CTV (prostate gland and basis of seminal vesicles) with a margin of 1 cm in all directions except posterior, where a 0.5 cm margin was specified. As organs at risk (OARs), bladder, rectum, and femoral heads were delineated. Dose prescription was 78 Gy in 2 Gy/fx to PTV.

Three oropharynx head and neck cases were contoured according to the clinical protocols; respective PTVs included CTV tumor and nodal CTVs with margin of 0.5 cm. Contralateral parotid gland, spinal cord, brainstem, and larynx were contoured as OARs. Dose prescription was 60 Gy in 2 Gy/fx to the PTV boost and 50 Gy to the nodal PTV.

Clinical plan acceptance criteria for all cases were specified as following: at least 95% of the PTV should be covered with 95% of the prescribed dose (as required by the institutional protocol), maximum dose to PTV should not exceed 115% of the prescribed dose, and the doses to OARs should not exceed the values recommended by QUANTEC group reports.^21^, ^22^, ^23^

### B. Treatment planning systems and equipment

The study was performed using two commercially available treatment planning systems (TPS) with VMAT optimization option. The first TPS, Monaco (Version 3.0.0), uses biologically constrained optimization for VMAT and IMRT; the other, Oncentra MasterPlan (OMP) (V 3.3 SP3; Nucletron, Veenendaal, The Netherlands), works only with dose‐volume objectives (DVO) and constraints. In order to reduce interplanner variability, the plans in both systems were created by the same physicist with three years of experience in using the software in a clinical routine. For both systems, 6 MV and 10 MV energies from Elekta Synergy linear accelerator (Elekta Ltd.) with MLCi (40 leaf pairs, 1 cm leaf width) were commissioned for VMAT and IMRT delivery.

### C. IMRT and VMAT treatment planning

When designing treatment planning study of different planning systems, in order to provide fair comparison of the results, as many parameters as possible should be the same. We used the same beam setup, beamlet size, and a dose calculation grid, and similar number of constraints in the prescription and segmentation parameters. This study design allows the assumption that observed dose differences in the planning result are mainly caused by the different optimization methods and cost function formalism in the respective systems. Additionally, application of the same parameters for the optimization and dose calculation ensures that the time needed to create a plan is approximately the same in both systems (20–30 min).

**Figure 1:**
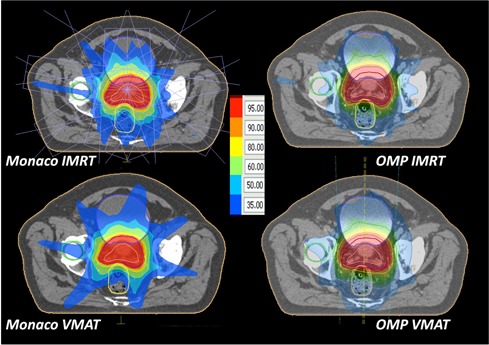
Representative dose distributions for prostate VMAT and IMRT plans in both treatment planning systems.

**Figure 2:**
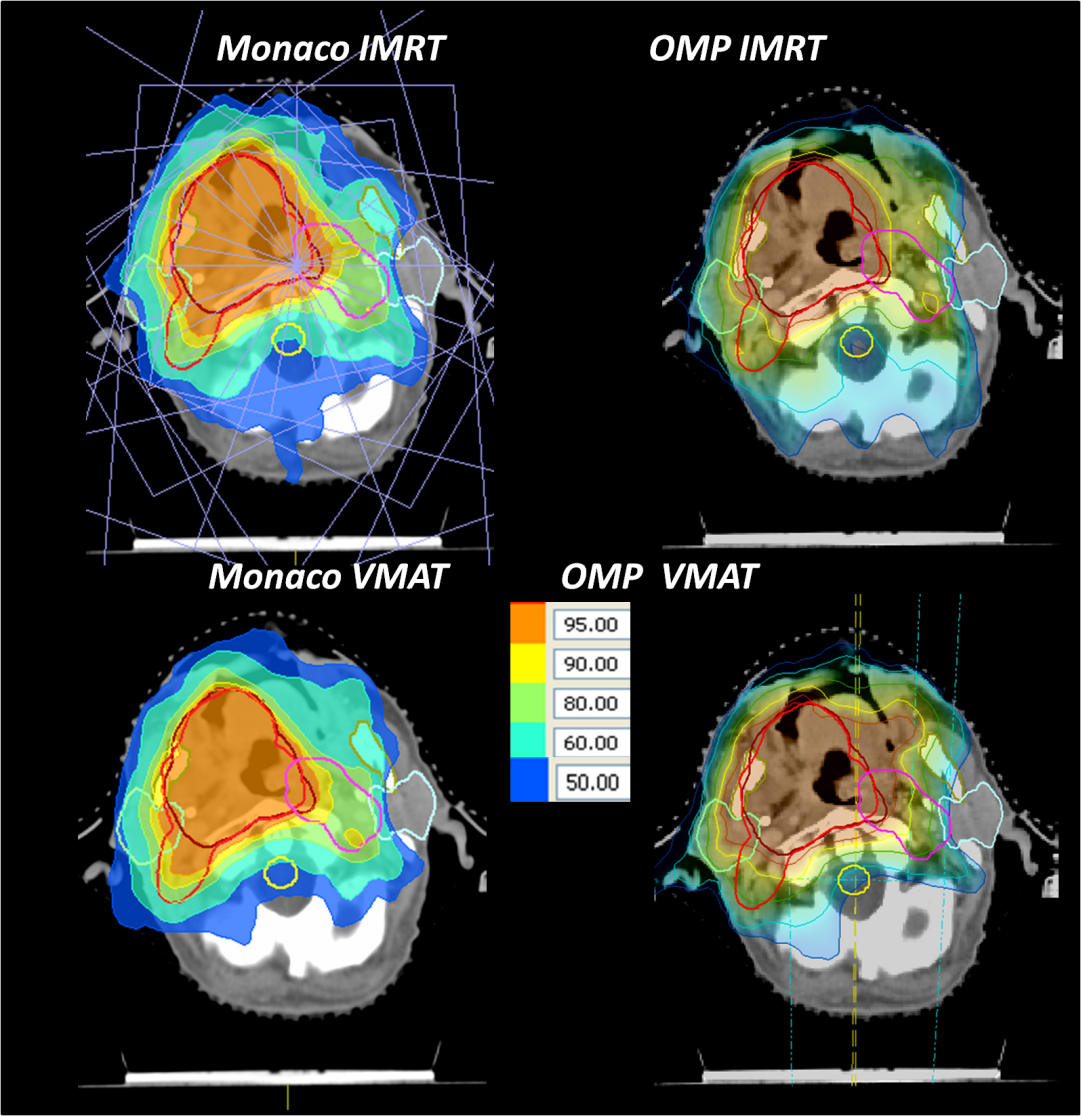
Representative dose distributions for head and neck VMAT and IMRT plans in both treatment planning systems.

For IMRT plans, nine equidistantly spaced beams (starting from gantry angle 0°, collimator 0°) with 6 MV and 10 MV energies were applied for head and neck and prostate cases, respectively. VMAT plans were created for the same photon energy as IMRT, using single arc with full 360° gantry rotation and collimator angle of 3°. For the purpose of the current comparison, as a starting point, an IMRT plan of the same quality in terms of DVH (see Figs. 1 and 2) was created in both systems and same prescription was used for IMRT and VMAT in both planning systems. Optimization parameters of initial plan leading to the clinically acceptable VMAT/IMRT prostate plan in Monaco and plan of the same quality in Oncentra MasterPlan (OMP) are listed in Table 1. Table 2 presents the similar overview of the parameters for representative head and neck plan.

IMRT and VMAT plans were optimized with the beamlet width of 0.4 cm. As in Oncentra MasterPlan, VMAT optimization starts for gantry angle spacing of 24°^(4)^ (this value is hard‐coded in the optimizer and defines initial sampling of the fluence maps according to the target aperture for the optimization), the same value was entered in Monaco as “Increment” in the “Beam Setup” dialog window, where a user provides an input on the treatment fields (number of beams, gantry and collimator angles, and selection of the treatment machine). The increment determines in how many sectors the initial full arc will be divided for the optimization process to generate fluence profiles at static gantry positions. First stage of the optimization was performed with fast pencil beam algorithm in both TPS, whereas final dose calculation was performed on 4 mm^3^ grid with Monte Carlo‐based (3% variance per segment/control point) and enhanced collapsed cone algorithms in Monaco and OMP, respectively. For prostate VMAT optimization and sequencing (including segment weight optimization), 91 control points were defined in both planning systems and minimal segment area was specified at 2 cm^2^ and minimal allowed number of monitor units (MU) per segment was set at four. For prostate IMRT plans, maximum 120 segments per plan were allowed in OMP, whereas in Monaco, to achieve same result, fluence smoothing parameter was set at 1 and “segment suppression factor” at value=3 to reduce number of segments to the same value approximately.

Following segmentation parameters were applied for H&N plans in both systems: 150 control points, minimal segment area $=4\;{\text{cm}}^{2}$, minimum 4 MU per segment. Settings limiting the number of segments in Monaco plans were set at the same values as for prostate cases.

As mentioned above, a biological formalism in terms of generalized EUD is used for the optimization functions in Monaco TPS. The main characteristic of the biological functions is that they are not point‐based (meaning that only one voxel above some threshold would violate the constraints), but rather region‐based through the use of EUD, which is the dose that would have the same biological effect as the actual inhomogeneous tissue dose distribution if the tissue coverage was spatially uniform. The generalized EUD (gEUD) can be formulated as:

(1) $$gEUD=(\sum_{i}v_{i}D_{i}^{a})1/a$$

where $v_{i}$ is the fractional organ volume receiving a dose $D_{i}$, and *a* is a tissue‐specific parameter that describes the volume effect. This formula allows us to consider tissue‐specific property into the planning process that cannot be done with dose‐volume‐based optimization. Serial cost function in Monaco TPS (used typically for the organs with serial behavior) requires to enter EUD, where value is similar to an acceptable maximum dose when the k value is large (e.g., 12), and is equivalent to the mean dose when the k value is equal to 1. Power Law Exponent (k) defines a volume effect parameter. In general, when a small k value is entered, a large volume effect is assumed. This means that low‐dose volumes and high‐dose volumes are approximately equally weighted. When a large k value is used, there is less tolerance for excessive damage to small volumes of the assigned structure. In this case, low‐dose volumes receive a very small weight related to high‐dose volumes. Parallel cost function requires that user assigns three parameters. The first parameter is the Reference Dose (EUD) whose value is analogous to the dose that is only acceptable for the majority of the structures and at which a clear dose response begins to show. The second parameter is the Mean Organ Damage to the structure in percent. The Mean Organ Damage is the biological equivalent to the fraction of the volume of the structure that can be sacrificed. The third parameter is the Power Law Exponent (k). This value changes the shape of the dose response curve and determines how responsive the structure is to the Reference Dose and Mean Organ Damage values entered. A higher k value translates to a steep dose response that often translates into a pronounced kink in the DVH curve.^13^, ^14^, ^15^, ^16^ Readers are referred to vendor‐provided manuals or training for more detailed descriptions about those cost functions.

**Table 1:** Summary of optimization prescription for prostate VMAT and IMRT cases in Monaco and Oncentra MasterPlan treatment planning systems. Dose optimization to OARs in Monaco was performed only with biological cost functions; optimization in OMP was done with dose‐volume objectives. EDU=equivalent uniform dose, k=parameter for penalty strength, regulating steepness of the DVH curve

*Structure*	*Monaco*	*Oncentra Master Plan*
PTV	EDU=79.5 Gy, cell sensitivity=0.5 quadratic overdose=80 Gy, dose excess=0.6 Gy	Min. dose 76.5 Gy to 98%V, weight 3000
		Max. dose 82 Gy to 3%V, weight 3000
		Max. dose 84 Gy, weight 3000
Rectum	Serial EUD=59 Gy,k=12 parallel reference dose=50 Gy,mean damage=50% K=3.5,shrink margin=0.5 cm	Max. dose 75 Gy to 10%V, weight 1000
		Max. dose 60 Gy to 20%V, weight 3000
		Max. dose 50 Gy to 35%V, weight 1000
Bladder	Serial EUD=65 Gy,k=10 parallel reference dose=55 Gy,mean damage=45% K=3,shrink margin=1 cm	Max. dose 75 Gy to 10%V, weight 1000
		Max. dose 65 Gy to 20%V, weight 1000
		Max. dose 50 Gy to 35%V, weight 1000
Femoral Heads	Serial EUD=35 Gy,k=5	Max. dose 50 Gy to 5%V, weight 1000
Body	quadratic overdose=74.1 Gy,dose excess=0.2 Gy quadratic overdose=40 Gy,dose excess=1 Gy,shrink margin=2 cm	Max. dose 74 Gy to 0.5%V, weight 3000
		Max. dose 25 Gy to 15%V, weight 3000

**Table 2:** Summary of optimization prescription for head and neck VMAT and IMRT cases in Monaco and Oncentra MasterPlan treatment planning systems. Dose optimization to OARs in Monaco was performed only with biological cost functions; optimization in OMP was done with dose‐volume objectives. EDU=equivalent uniform dose,k=parameter for penalty strength, regulating steepness of the DVH curve

*Structure*	*Monaco*	*Oncentra MasterPlan*
PTV Boost 60 Gy	Target EUD=61Gy,cell sensitivity=0.5 quadratic overdose=62 Gy,dose excess=0.8 Gy	Min. dose 59 Gy to 98%V, weight 3000
		Max. dose 63 Gy to 3%V, weight 3000
		Max. dose 66 Gy, weight 3000
PTV Nodes 50 Gy	Target EUD=51 Gy,cell sensitivity=0.5 quadratic overdose=60 Gy,dose excess=1 Gy quadratic overdose=52 Gy,dose excess=1 Gy, shrink margin=1 cm	Min. dose 49 Gy to 98%V, weight 3000
		Max. dose 55 Gy to 3%V, weight 3000
Cord	Serial EUD=40 Gy,k=16	Max. dose 35 Gy to 2%V, weight 3000
Brainstem	Serial EUD=40 Gy,k=12	Max. dose 40 Gy, weight 3000
Parotid Gland	parallel reference dose=25 Gy, mean damage=45%,k=3.5	Max. dose 22 Gy to 33%V, weight 3000
		Max. dose 55 Gy, weight 3000
Body	quadratic overdose=60 Gy,dose excess=0.3 Gy quadratic overdose=40 Gy,dose excess=1 Gy,shrink margin=0.8 cm quadratic overdose=30 Gy,dose excess=0.8 Gy,shrink margin=2.4 cm	Min. dose 55 Gy to 2%V, weight 3000
		Max. dose 20 Gy to 22%V, weight 3000

### D. Plan evaluation and construction of Pareto fronts

Evaluation of the plans was performed according to the recent ICRU Report 83 recommendations;^(24)^ additionally, relevant dose‐volume parameters from QUANTEC reports were assessed. For PTVs, minimum, maximum, and median doses were reported. As surrogates for minimum and maximum dose, the dose to 98% and 2% of the target volume were evaluated. In addition, we reported tumor coverage as percentage of the respective PTV volume covered with 95% of the prescribed dose for all VMAT and IMRT plans. The target conformity index (CI), as proposed by Paddick,^(25)^ was evaluated for the PTV volume covered with 95% isodose:

(2) $$CI=\frac{{\left(V_{PTV}^{95\%D}\right)}^{2}}{V_{PTV}\cdot V_{95\%D}}$$

where $V_{PTV}$ stands for the PTV volume in ccm, $V_{95\%D}$ refers to the total volume covered with 95% isodose line, and ${V_{PTV}}^{95\%D}$ corresponds to the part of the PTV volume covered with 95% isodose.

In CI, target homogeneity index (HI), defined as

(3) $$HI=\frac{D_{2\%}-D_{98\%}}{D_{medium}}$$

was calculated.

For OARs maximum dose (as well taken as dose to 2% of the OAR volume), median or mean doses were used for comparison. In prostate cases rectum volume covered by 60 Gy $\left(V_{60\text{Gy}}\right)$ and for bladder $V_{65\text{Gy}}$ were assessed. In H&N cases, the following OAR dose‐volume indices were evaluated: $D_{2\%}$ for spinal cord and brainstem, and mean dose for contralateral parotid gland. In all VMAT and IMRT plans, volume of the patient covered with 5 Gy was recorded.

Again, in order to reduce the error caused by different methods of DVH calculation in both TPS, calculated dose was imported in Monaco TPS and DVH curves and parameters were calculated with 1 mm resolution and 0.1 Gy dose bins. Number of segments (control points) and monitor units (MU) were reported for each plan, as well.

A treatment plan is Pareto optimal for a given set of objectives and constraints if there is no other feasible treatment plan that is at least as good in all the objectives and strictly better in at least one objective. A Pareto front is constituted by the Pareto optimal solutions and comparison of the sampled Pareto fronts, rather than individual treatment plans, gives better picture about abilities of the TPS to achieve required planning objectives.

Pareto fronts were created by adjusting the optimization constraints of the initial clinical acceptable plan (as shown in Tables 1 and 2) for one of the organs at risk, while keeping other parameters constant. As Monaco employs the biological EUD‐based formalism of the cost functions for OAR optimizations, we have chosen to vary EUD value in Serial or “Mean Organ damage %” value in parallel cost function, whereas in OMP, one DVO value was changed. For each case, 15 plans were optimized for the front sampling, until the plan acceptance criteria in terms of PTV coverage were violated.

In prostate cases, for rectum we consecutively lowered Serial EUD value or DVO “Max dose to 20% of the volume” by 0.5 Gy in Monaco and OMP, respectively. Pareto fronts were sampled based on the clinical acceptance criteria for the PTV coverage and relative rectum volume receiving 60 Gy. Assuming that biologically constrained cost function works on the whole DVH curve opposite to the DVO optimization concept, median dose to rectum was evaluated, as well. In head and neck cases, we changed for parotid gland volume percentage “Mean Organ damage %” value in parallel cost function in Monaco in 3% steps, starting from the parameters in Table 2. In OMP, DVO for percentage of volume receiving 25 Gy was adjusted. Pareto fronts were constructed for the mean dose to parotid gland versus PTV coverage by 95% isodose.

## III. RESULTS

### A. Prostate

**Figure 3:**
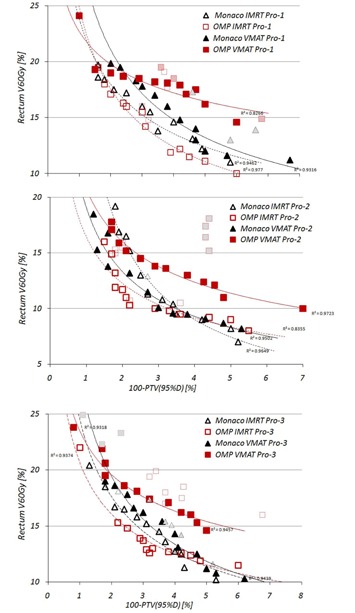
Pareto fronts for prostate cases. Symbols with dotted contour and light grey filling indicate non‐Pareto optimal solutions, provided by optimizer.

Variation of the dose‐volume constraint for rectum, together with PTV coverage, is reported in Table 3. Pareto fronts, based on these parameters for all three prostate cases, are shown in Fig. 3. In case of OMP, quality of IMRT plans in terms of rectum sparing and PTV coverage was superior to VMAT solutions for all three cases. For Monaco, this trend was confirmed for Pro‐1 case, whereas for Pro‐2 and Pro‐3 cases, VMAT and IMRT plans resulted in very similar plan quality.

In the intersystem comparison for IMRT plans, for the cases with significant dose reduction to rectum and still above PTV coverage, over 95% of both systems generated very similar solutions. With regard to VMAT plans, optimization solutions from Monaco were better for rectum sparing and PTV coverage than OMP, as shown by solid black trend lines for all three cases.

Table 4 shows the variation for the dose‐volume indices observed while creating Pareto fronts for prostate cases. Front sampling process did not influence much median and maximum PTV doses, as shown by small standard deviations in the Table 3. The influence on the plan conformity and homogeneity was minimal, as well. Both TPS demonstrated very similar homogeneity in VMAT and IMRT plans, except OMP VMAT plans for Pro‐2 case. This can be explained by large intersection between PTV and rectum volumes. Plan conformity indices were similar for IMRT and VMAT plans, as well, but conformity of the Monaco solutions was much better (10% and more) compared to OMP.

For the rectum and bladder, choice of the delivery technique and TPS had almost no influence for $D_{2\%}$ in rectum and bladder. With regard to the median dose to rectum, in both systems VMAT plans resulted in 2%–5% lower values compared to IMRT. However, while evaluating $D_{\text{median}}$ and $V_{65\text{Gy}}$ of the bladder, very large difference was demonstrated for EUD‐based optimization compared to OMP. Median doses to bladder were 10% to 20% lower in case of Monaco in VMAT and IMRT plans (see Table 4). Volume of the nontarget tissue receiving 5 Gy and more was quite different between both delivery techniques and planning systems. In case of Monaco, VMAT plans resulted in 300–500 ccm larger V5Gy. For OMP only in Pro‐1 case, VMAT plans resulted in larger 5 Gy volume; for two other cases VMAT plans resulted in over 800 ccm lower V5Gy. In the intersystem analysis, almost no difference in 5 Gy volume was demonstrated in VMAT plans, whereas Monaco IMRT plans showed lowest values in all cases.

The variation of the parameters influencing the delivery efficiency is presented in the Table 5. The number of segments in Monaco IMRT plans, as well as number of MUs, was constantly higher compared to OMP. Due to specifics of VMAT sequencer in Monaco with variable number of control points per arc, solutions with either more or less controls points, compared to OMP, are presented and similar to IMRT cases show more MUs per arc. The difference in MU between VMAT and IMRT plans was below 100 MU in both systems for all three cases.

**Table 3:** Data for generation of Pareto fronts. Range of PTV coverage, rectal volume constraints, and mean parotid gland doses, for prostate and head and neck cases, respectively

*TPS/Plan*	*Case*	*PTV* $V_{D95\%}$	*Rectum* $V_{60Gy}(\%)$	*Case*	*PTV 50 Gy* $V_{D95\%}$	*Parotid* $D_{mean}[Gy]$
Monaco IMRT	Pro‐1	94.2–98.4	11.0–19.7	HN1	97.5–99.2	17.9–25.2
Monaco VMAT		92.3–97.7	11.2–19.5		89.1–98.0	17.1–24.0
OMP IMRT		94.9–97.0	11.9–19.1		92.5–98.1	15.1–23.8
OMP VMAT		93.2–99.0	14.6–24.1		94.6–97.0	15.6–19.8
Monaco IMRT	Pro‐2	96.5–98.2	9.5–19.2	HN2	94.9–97.5	17.0–24.5
Monaco VMAT		93.8–98.7	9.5–16.8		93.1–98.0	17.7–25.3
OMP IMRT		96.4–98.3	10.3–15.9		96.8–97.4	18.2–23.9
OMP VMAT		95.6–98.3	15.2–18.1		87.2–95.8	18.6–24.1
Monaco IMRT	Pro‐3	94.7–98.7	10.2–20.4	HN3	90.9–97.6	16.4–25.3
Monaco VMAT		93.8–97.5	10.3–18.4		82.7–97.9	16.5–24.4
OMP IMRT		93.2–97.1	13.5–16.4		97.0–98.4	17.7–25.1
OMP VMAT		97.7–99.2	19.5–24.9		88.0–97.0	14.5–22.3

**Table 4:** Overview of the dose‐volume parameters for prostate plans. Data presented as mean ± standard deviation for all created plans

		*PTV*			*Recturn*		*Bladder*			*Body*		
*Case*	*TPS/Plan*	$D_{median}$	$D_{2\%}$	$D_{98\%}$	$D_{2\%}$	$D_{median}$	$D_{2\%}$	$D_{median}$	$V_{65Gy}$	$V_{5Gy}$ *(ccm)*	*HI*	*CI*
Pro‐1	Monaco IMRT	102.5±0.3	105.5±0.1	93.8±1.3	100.4±0.8	49.3±5.3	101.8±0.5	25.7±1.2	10.8±0.5	5098±28	0.11±0.01	0.84±0.01
	Monaco VMAT	101.5±1.3	105.1±0.3	92.8±1.3	98.6±0.9	47.6±5.3	101.4±0.4	24.9±0.5	10.4±0.2	5498±23	0.12±0.02	0.85±0.01
	OMP IMRT	100.8±0.5	105.2±0.5	93.0±0.7	97.6±0.5	54.3±3.6	101.6±1.0	32.6±1.0	20.0±0.6	5740±18	0.12±0.01	0.69±0.01
	OMP VMAT	100.4±1.2	104.6±2.5	93.4±2.4	99.4±1.2	47.1±3.7	101.8±0.6	34.6±0.6	19.9±0.5	5999±14	0.11±0.03	0.58±0.01
Pro‐2	Monaco IMRT	102.0±0.2	105.0±0.1	94.5±0.6	97.0±1.7	54.0±4.7	102.3±0.4	41.9±1.2	17.5±0.2	6716±79	0.10±0.01	0.91±0.01
	Monaco VMAT	101.2±0.6	104.8±0.1	94.7±1.0	96.5±2.2	49.9±3.8	102.4±0.9	38.9±0.7	16.8±0.3	7369±33	0.10±0.01	0.90±0.01
	OMP IMRT	100.4±0.3	105.0±0.7	95.0±0.5	96.1±0.7	56.1±2.4	102.4±1.0	60.0±0.7	29.7±0.8	8206±40	0.10±0.01	0.71±0.02
	OMP VMAT	100.8±1.1	112.3±4.3	94.3±0.9	97.9±1.3	45.7±2.5	105.8±1.3	49.5±2.0	25.2±1.2	7395±97	0.18±0.05	0.78±0.02
Pro‐3	Monaco IMRT	102.5±0.4	105.5±0.2	93.0±2.0	99.2±1.1	48.0±5.6	100.3±0.3	25.1±1.3	6.3±0.3	5476±42	0.12±0.02	0.88±0.01
	Monaco VMAT	101.6±0.3	105.4±0.2	92.6±1.5	97.8±1.2	46.6±5.7	99.5±0.5	25.3±0.5	6.1±0.2	5715±29	0.13±0.02	0.88±0.02
	OMP IMRT	99.4±0.5	104.0±0.6	93.0±0.5	96.6±0.9	55.5±3.0	99.2±0.6	35.4±0.4	11.3±0.2	6635±31	0.11±0.01	0.69±0.02
	OMP VMAT	101.5±1.3	107.1±3.2	95.5±0.8	101.3±0.2	49.0±4.0	100.5±1.1	25.4±0.5	10.2±0.7	5708±56	0.11±0.04	0.75±0.01

**Table 5:** Delivery efficiency of prostate and head and neck plans. Number of segments (control points) is reported as range; for MUs, mean ± standard deviation is reported

		*Monaco*		*OMP*	
*Case*		*IMRT*	*VMAT*	*IMRT*	*VMAT*
Pro‐1	Segments/CP	55–69	86–111	40–54	91
	MU	575.4±58.9	615.1±41.1	423.3.1±21.9	380.5±9.4
		(507.6–667.9)	(558.7–682.9)	(395.7–467.3)	(369.2–394.3)
Pro‐2	Segments/CP	69–82	106–136	50–65	91
	MU	671.8±45.9	732.3±33.1	484.5±40.4	552.3±4.6
		(612.9–719)	(683.2–788.3)	(433.3–571.1)	(544.7–558.6)
Pro‐3	Segments/CP	64–74	98–123	37–57	91
	MU	618.7±51.2	680.7±46.5	437.9±20.8	405.9±16.1
		(556.4–700.8)	(605–747)	(389.9–469.9)	(369.4–421)
HN1	Segments/CP	78–95	125–136	88–101	91
	MU	653.9±56.4	739.9±8.5	743.8±45.0	452.6±23.8
		(564.2–719.4)	(728.7–758.1)	(676.7–808.6)	(427–501)
HN2	Segments/CP	104–113	127–133	105–116	91
	MU	785.8±35.8	702.8±39.0	769.2±31.0	630.8±55.8
		(743.7–830.8)	(639.4–747.2)	(720.6–815.8)	(551.1–717.4)
HN3	Segments/CP	91–100	112–127	98–114	91
	MU	671.3±37.6	723.4±26.5	815.2±31.0	645.1±106.4
		(611.6–728.4)	(683.3–749.2)	(753–846.9)	(458.5–775.1)

### B. Head and neck

**Figure 4:**
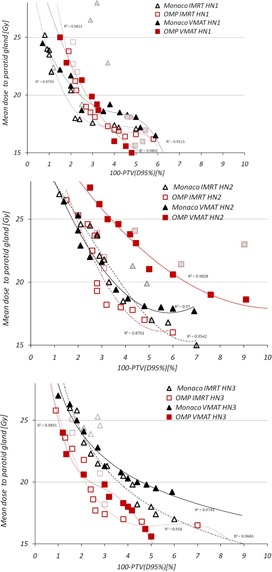
Pareto fronts for head and neck cases. Symbols with dotted contour and light grey filling indicate non‐Pareto optimal solutions, provided by optimizer.

Variation of the mean dose constraint to parotid gland, together with PTV coverage, is reported in Table 3. Pareto fronts, based on these parameters for all three head and neck cases, are shown in Fig. 4. In both systems, IMRT solutions were superior to VMAT in regions with excessive parotid sparing. Intersystem comparison showed very similar IMRT plan quality for HN1 and HN2 cases; in a third case, OMP solutions were better than Monaco.

Table 6 shows the variation for the dose‐volume indices observed while creating Pareto fronts for head and neck cases. Similar to the prostate cases, Pareto front sampling process did not influence much median and maximum PTV doses; however, coverage of the PTV boost was sometimes compromised. $D_{2\%}$ for spinal cord and brainstem were either better or similar for IMRT plans in both systems. Volume of normal tissue receiving 5 Gy or more was very similar between both systems and delivery techniques, again with lowest values in Monaco IMRT plans. Solutions obtained with Monaco IMRT planning resulted in best target homogeneity, as shown in Table 6. Plan conformity was quite similar in both TPS, with variations around 5% between different delivery techniques.

Delivery efficiency of IMRT plans in terms of segments and monitor units, as shown in Table 5, was slightly better for Monaco compared to OMP. Difference between VMAT and IMRT MUs was, again, very small in case of Monaco, whereas for OMP, approximately 250 MU less was needed for VMAT plans.

**Table 6:** Overview of the dose‐volume parameters for head and neck plans. Data presented as mean ± standard deviation for all created plans

		*PTV 60 Gy Bost*				*PTV 50 Gy*			*Spinal Cord*	*Brainstem*	*Body*		
*Case*	*TPS/Plan*	$V_{D95\%}$	$D_{median}$	$D_{2\%}$	$D_{98\%}$	$D_{median}$	$D_{2\%}$	$D_{98\%}$	$D_{2\%}$ *(Gy)*	$D_{2\%}$ *(Gy)*	$V_{5Gy}$ *(ccm)*	*HI*	*CI*
HN1	Monaco IMRT	99.3±0.1	103.3±0.2	107.0±0.1	96.8±0.2	104.4±0.4	110.1±0.2	95.8±1.0	431±1	36.6±0.9	5122±29	0.10±0.01	0.69±0.02
	Monaco VMAT	95.9±1.6	102.1±0.3	107.5±0.2	93.6±0.9	103.1±1.1	113.1±0.2	92.6±2.3	44.1±0.9	40.0±1.8	5099±18	0.14±0.01	0.74±0.06
	OMP IMRT	95.4±0.8	100.5±0.4	105.6±0.8	92.2±0.7	104.0±0.8	115.2±2.1	94.1±1.6	34.9±1.0	31.2±0.4	5294±10	0.13±0.01	0.61±0.02
	OMP VMAT	97.2±0.6	103.2±0.3	108.7±0.3	94.2±0.5	103.9±0.4	110.2±0.3	89.9±2.0	35.1±0.5	34.1±0.9	4860±75	0.14±0.01	0.66±0.02
HN2	Monaco IMRT	98.7±0.5	103.6±0.2	107.7±0.1	96.3±0.9	106.1±0.5	123.6±0.4	91.6±2.2	42.3±2.1	43.3±1.6	5764±38	0.11±0.01	0.72±0.02
	Monaco VMAT	92.9±5.4	102.6±2.2	110.0±1.8	91.3±3.7	100.1±6.7	120.1±5.9	83.7±6.8	42.8±1.1	46.3±1.6	5897±47	0.18±0.03	0.61±0.02
	OMP IMRT	95.9±0.2	103.5±0.2	108.4±0.2	92.2±0.2	104.4±0.3	116.9±0.4	92.2±1.2	33.4±0.2	37.7±0.9	5884±8	0.16±0.01	0.71±0.06
	OMP VMAT	95.9±1.7	102.3±0.6	108.3±0.8	92.5±1.6	103.0±0.9	116.7±3.4	88.6±3.7	33.5±1.9	37.2±1.1	5521±103	0.15±0.02	0.65±0.03
HN3	Monaco IMRT	99.5±0.6	103.2±0.5	107.1±0.2	97.4±1.1	105.0±0.7	124.8±0.8	92.4±2.3	39.7±1.4	41.3±2.1	5618±41	0.09±0.01	0.74±0.02
	Monaco VMAT	97.0±2.8	102.5±0.5	107.3±0.1	94.5±1.9	104.3±1.4	125.0±1.4	91.8±3.2	40.1±2.4	40.6±2.0	5701±44	0.13±0.02	0.77±0.02
	OMP IMRT	96.3±0.2	102.1±0.3	107.2±0.5	93.4±0.3	104.9±0.7	121.1±0.6	94.3±1.7	35.5±0.6	40.3±0.4	5953±11	0.14±0.01	0.73±0.01
	OMP VMAT	92.5±3.8	102.1±0.6	109.4±1.4	91.2±2.3	103.5±0.7	121.9±1.1	87.2±4.3	34.3±0.9	39.3±0.5	5689±180	0.18±0.03	0.69±0.05

## IV. DISCUSSION

Pareto fronts represent valid and convenient tool to explore potential of the optimizer in different planning systems to achieve desirable balance between doses to target and OARs. From a clinical point of view, data obtained with Pareto fronts can provide an estimate of the potential reduction of OAR dose and, therefore, reduce complication probability. The advantage of using biological models in treatment planning is that all the voxels present in the region of interest are playing a role in the optimization process instead of the few outlier voxels that receive a disproportionate optimization weight due to point‐based constraint violations. Biological cost functions offer more control over the dose distribution than physical cost functions, and a smaller number of them is required to fully shape the dose distribution.

The question of which treatment technique (IMRT or VMAT) is preferable in terms of target coverage and organ sparing was discussed controversially in the literature.^7^, ^8^, ^9^, ^10^, ^11^, ^12^, ^26^ Comparison with the published data was difficult, because these studies employ different treatment planning systems and do not use Pareto fronts as plan analysis tool. In case of Oncentra MasterPlan, published data suggest better target coverage and OAR sparing for single‐arc VMAT for prostate cancer compared to IMRT, whereas for head and neck, use of dual‐arc was recommended to achieve results comparable with IMRT.^4^, ^27^

In the situation where resources are very limited (number of workstations for planning, large patient throughput), a choice of the TPS is important to avoid delays in the clinical routine during planning process and also to be able to provide advantageous planning solutions for the given clinical case.

As can be seen in our study, Pareto fronts for prostate cases show that single‐arc VMAT plans are worse compared to nine‐field IMRT plans for Oncentra Master Plan, whereas for Monaco TPS this difference in plan quality was minimal. With regard to other parameters used for plan evaluation, in the case of prostate plans, homogeneity and conformity indices, together with median bladder dose and lower 5 Gy volume, indicate clinically better solutions created with Monaco; however, this benefit is slightly compromised by the reduced delivery efficiency of IMRT plans. Taking all factors and delivery efficiency into account, VMAT delivery based on Monaco‐optimized prostate plans was selected as preferable option in our institution.

For head and neck case NH2, where very strong modulation was required due to proximity of the OARs and large overlap of the parotid gland with PTV, VMAT plans done with Oncentra were inferior compared to IMRT solutions. This confirms that, for cases where strong modulation is required, dual‐arc VMAT or IMRT would be preferable option. In two other cases, IMRT and VMAT solutions of OMP were quite similar. In Monaco system, both IMRT and VMAT plans were showing same plan quality, with results close to IMRT plans done with Oncentra, except HN3 case. In our opinion, here good solution could be possibly achieved with Monaco TPS if we would allow more control points per arc and, consequently, more MUs to achieve similar modulation. In general, for all head and neck cases, Monaco provided better target dose homogeneity and conformity and only small increase of the dose to spinal cord and brainstem within clinically acceptable range. Based on these findings, we prefer use of IMRT in Oncentra or Monaco TPS when strong modulation is required in complex head and neck cases, and VMAT in less complex situations where planning aims can be achieved with single‐arc VMAT. Due to fast planning process (DVH control by biological cost functions) and ability to create class solutions (plan templates) with Monaco system, VMAT planning is less time‐consuming. With regard to the efficiency of plan delivery, more conformal dose distributions obtained with Monaco are achieved by the system due to the higher degree of modulation in the plans and, consequently, require more MUs.

In our opinion, possible expected gain in doses to OARs in highly complex plans with use of biological cost functions in Monaco was compromised by VMAT sequencer in this version of TPS, as also noticed in the paper by Nevelsky at al.^(28)^ Hence speed of the treatment delivery is reduced with VMAT by approximately 60%.^2^, ^4^, ^5^, ^10^ The data obtained in this study show that single‐arc VMAT represents valid alternative to static nine‐beam IMRT in both prostate and head and neck cases.

As was pointed out, it is difficult to design a treatment planning study for systems due to the differences in the implementation of optimization and sequencing algorithms. Comparison between optimization results done before sequencing would provide better insight on the advantages of the biological cost functions; however, without a creation of deliverable plans, these results would have little practical value. Sequencing for VMAT in Monaco and Oncentra and inability to change flexibly number of control points per arc in OMP can be considered as a major limitation of the system. On the other hand, absence of segment shape optimization in version 3.00 of Monaco produced large difference between fluence obtained in the first stage of the optimization and final result. This feature was implemented in later releases and expected to improve further MU efficiency of the Monaco VMAT plans.

## V. CONCLUSIONS

It was found in this study that for the prostate cases VMAT plans generated with Monaco TPS showed the same dosimetric quality as nine‐beam IMRT plans. In the Oncentra system, the difference was much more pronounced in favor of IMRT solutions. Compared with Oncentra system, Monaco VMAT plans also reduced median dose to bladder and showed a better target conformity due to the use of biological cost functions. For head and neck cases with complex anatomy where strong modulation is desired, static beam IMRT plans or VMAT plans with large number of control points per arc (120 or above) and dual‐arc VMAT technique would be preferred in both treatment planning systems. Limitations of the sequencer reduced the potential advantage of the biological optimization for head and neck plans and produced similar results in intersystem comparison.

## Supporting information

Supplementary Material

## References

[1] Yu CX , Tang G . Intensity‐modulated arc therapy: principles, technologies and clinical implementation. Phys Med Biol. 2011;56:R31–R54. 21297245 10.1088/0031-9155/56/5/R01

[2] Bedford JL . Treatment planning for volumetric modulated arc therapy. Med Phys. 2009;36(11):5128–38. 19994523 10.1118/1.3240488

[3] Rao M , Yang W , Chen F , et al. Comparison of Elekta VMAT with helical tomotherapy and fixed field IMRT: plan quality, delivery efficiency and accuracy. Med Phys. 2010;37(3):1350–59. 20384272 10.1118/1.3326965

[4] Alvarez‐Moret J , Pohl F , Koelbl O , Dobler B . Evaluation of volumetric modulated arc therapy (VMAT) with Oncentra MasterPlan for the treatment of head and neck cancer. Radiat Oncol. 2010;5:110. 21092163 10.1186/1748-717X-5-110 PMC2998512

[5] Bertelsen A , Hansen CR , Johansen J , Brink C . Single arc volumetric modulated arc therapy of head and neck cancer. Radiother Oncol. 2010;95(2):142–48. 20188427 10.1016/j.radonc.2010.01.011

[6] Pesce G , Clivio A , Cozzi L , et al. Early clinical experience of radiotherapy of prostate cancer with volumetric modulated arc therapy. Radiat Oncol. 2010;5:54. 20550722 10.1186/1748-717X-5-54 PMC2902493

[7] Clemente S , Wu BB , Sanguineti G , et al. SmartArc‐based volumetric modulated arc therapy for oropharyngeal cancer: a dosimetric comparison with both intensity‐modulated radiation therapy and helical tomotherapy. Int J Rad Oncol Biol Phys. 2011;80(4):1248–55. 10.1016/j.ijrobp.2010.08.007 20947268

[8] Tang G , Phil M , Earl MA , Wang C , Mohiuddin MM , Yu CX . Comparing radiation treatments using intensity‐modulated beams, multiple arcs, and single arcs. Int J Rad Oncol Biol Phys. 2010;76(5):1554–62. 10.1016/j.ijrobp.2009.04.003 PMC2846542 20338482

[9] Davidson MT , Blake SJ , Batchelar DL , Cheung P , Mah K . Assessing the role of volumetric modulated arc therapy (VMAT) relative to IMRT and helical tomotherapy in the management of localized, locally advanced, and postoperative prostate cancer. Int J Rad Oncol Biol Phys. 2011;80(5):1550–58. 10.1016/j.ijrobp.2010.10.024 21543164

[10] Wiezorek T , Brachwitz T , Georg D , et al. Rotational IMRT techniques compared to fixed gantry IMRT and Tomotherapy: multi‐institutional planning study for head‐and‐neck cases. Radiat Oncol. 2011;6:20. 21338501 10.1186/1748-717X-6-20 PMC3050734

[11] Wolff D , Stieler F , Welzel G , et al. Volumetric modulated arc therapy (VMAT) vs. serial tomotherapy, step‐and‐shoot IMRT and 3D‐conformal RT for treatment of prostate cancer. Radiother Oncol. 2009;93(2):226–33. 19765846 10.1016/j.radonc.2009.08.011

[12] Palma D , Vollans E , James K , et al. Volumetric modulated arc therapy for delivery of prostate radiotherapy: complication with intensity‐modulated radiotherapy and three‐dimensional conformal radiotherapy. Int J Rad Oncol Biol Phys. 2008;72(4):996–1001. 10.1016/j.ijrobp.2008.02.047 18455326

[13] Alber M and Nuesslin F . An objective function for radiation treatment optimization based on local biological measures. Phys Med Biol. 1999;44(2):479–93. 10070796 10.1088/0031-9155/44/2/014

[14] Thieke C , Bortfeld T , Niemierko A , Nill S . From physical dose constraints to equivalent uniform dose constraints in inverse radiotherapy planning. Med Phys. 2003;30(9):2332–39. 14528955 10.1118/1.1598852

[15] Semenenko VA , Reitz B , Day E , et al: Evaluation of a commercial biologically based IMRT treatment planning system. Med Phys 2008, 35 (12):5851–5860. 19175141 10.1118/1.3013556

[16] Qi XS , Semenenko VA , Li XA . Improved critical structure sparing with biologically based IMRT optimization. Med Phys. 2009;36(5):1790–99. 19544798 10.1118/1.3116775

[17] Mihaylov IB , Fatyga M , Bzdusek K , Gardner K , Moros EG . Biological optimization in volumetric modulated arc radiotherapy for prostate carcinoma. Int J Rad Oncol Biol Phys. 2012;82(3):1292–98. 10.1016/j.ijrobp.2010.06.020 21570214

[18] Ottosson RO , Engstrom PE , Sjostrom D , et al. The feasibility of using Pareto fronts for comparison of treatment planning systems and delivery techniques. Acta Oncol. 2009;48(2):233–37. 18752085 10.1080/02841860802251559

[19] Ottosson RO , Karlsson A , Behrens CF . Pareto front analysis of 6 and 15 MV dynamic IMRT for lung cancer using pencil beam, AAA and Monte Carlo. Phys Med Biol. 2010;55(16):4521–33. 20668346 10.1088/0031-9155/55/16/S07

[20] Petersson K , Ceberg C , Engstrom P , Benedek H , Nilsson P , Knoos T . Conversion of helical tomotherapy plans to step‐and‐shoot IMRT plans—Pareto front evaluation of plans from a new treatment planning system. Med Phys. 2011;38(6):3130–38. 21815387 10.1118/1.3592934

[21] Bentzen SM , Constine LS , Deasy JO , et al. Quantitative analyses of normal tissue effects in the clinic (QUANTEC): an introduction to the scientific issues. Int J Radiat Oncol Biol Phys. 2010;76(3 Suppl):S1–S160. 20171515 10.1016/j.ijrobp.2009.09.040 PMC3431964

[22] Viswanathan AN , Yorke ED , Marks LB , Eifel PJ , Shipley WU . Radiation dose‐volume effects of the urinary bladder. Int J Radiat Oncol Biol Phys. 2010;76(3 Suppl):S116–S122. 20171505 10.1016/j.ijrobp.2009.02.090 PMC3587780

[23] Michalski J , Gay H , Jackson A , Tucker SL , Deasy JO . Radiation dose‐volume effects in radiation‐induced rectal injury. Int J Radiat Oncol Biol Phys. 2010;76(3 Suppl):S123–S129. 20171506 10.1016/j.ijrobp.2009.03.078 PMC3319467

[24] ICRU . Prescribing, recording, and reporting photon‐beam intensity‐modulated radiation therapy (IMRT). ICRU Report 83. J ICRU. 2010;10(1).

[25] Paddick I . A simple scoring ratio to index the conformity of radiosurgical treatment plans. Technical note. J Neurosurg. 2000;93(Suppl 3):219–22. 11143252 10.3171/jns.2000.93.supplement

[26] Doornaert P , Verbakel WF , Bieker M , Slotman BJ , Senan S . RapidArc planning and delivery in patients with locally advanced head‐and‐neck cancer undergoing chemoradiotherapy. Int J Radiat Oncol Biol Phys. 2011;79(2):429–35. 20421159 10.1016/j.ijrobp.2009.11.014

[27] Dobler B , Weidner K , Koelbl O . Application of volumetric modulated arc therapy (VMAT) in a dual‐vendor environment. Radiat Oncol. 2010;5:95. 20973977 10.1186/1748-717X-5-95 PMC2987940

[28] Nevelsky A , Ieumwananonthachai N , Kaidar‐Person O , et al. Hippocampal‐sparing whole‐brain radiotherapy using Elekta equipment. J Appl Clin Med Phys. 2013;14(3):4205. 23652251 10.1120/jacmp.v14i3.4205 PMC5714429

