# Supplementary material for: Efficiency of biological versus physical optimization for single‐arc VMAT for prostate and head and neck cases
Source: J Appl Clin Med Phys. 2014 Jul 8;15(4):39–53. doi: 10.1120/jacmp.v15i4.4514 (PMC5875496; doi:10.1120/jacmp.v15i4.4514)
Supplement: Supplementary file 1 — Supplementary Material [file ACM2-15-39-s001.doc]

# Efficiency of biological vs. physical optimization for single-arc VMAT for prostate and head-and-neck cases

***Short running title:*** *Biological vs. physical optimization for single-arc VMAT*

***ABSTRACT***

***Background:*** The aim of this work was to compare different approaches to VMAT optimization (biological vs. physical DVH-based) in two commercial treatment planning systems (TPS) for head-and-neck and prostate cases, using Pareto fronts. VMAT vs. IMRT Pareto front comparison was additionally performed in order to benchmark the optimizer efficiency and VMAT plan quality for each TPS.

***Methods:*** Three prostate and three head and neck cancer patients were selected for 9 beam IMRT and single arc VMAT planning in Monaco 3.00 and Oncentra MasterPlan 3.3 planning systems. Pareto fronts for prostate cases were constructed based on PTV coverage by 95% isodose and volume of rectum receiving 60 Gy or more. For head-and-neck cases PTV coverage by the same isodose and mean dose to parotid gland were used for the construction of Pareto fronts. DVH analysis was performed together with evaluation of planning and delivery efficiency for all the plans.

***Results:*** In the intersystem comparison for prostate plans, Monaco generated very similar IMRT and VMAT solutions. Quality of Monaco VMAT plans was superior compared to Oncentra in terms of conformity, homogeneity and lower median dose to bladder due to biological formalism of optimization cost functions. For the head-and-neck cases IMRT and VMAT plans were similar in both systems except the case where a very strong modulation was required. In this situation single arc VMAT plan generated with OMP was inferior compared to IMRT. VMAT OMP solutions were similar to Monaco or slightly better for two less modulated head-and-neck cases. However, this advantage was achieved on the cost of lower conformity and homogeneity of the Oncentra VMAT plans.

***Conclusions:*** IMRT and VMAT solutions generated by Monaco were very similar for both prostate and head-and –neck cases. Oncentra system shows bigger difference and use of the dual-arc VMAT would be recommended to achieve the same plan quality as 9 fields IMRT. Biological optimization seems beneficial in terms of plan conformity and homogeneity and allowed achieving lower OAR doses for prostate cases. In complex anatomical situations represented by head-and-neck cases sequencing algorithm in Monaco imposed limitations on VMAT plan quality in the intersystem comparison.

***Keywords:*** *VMAT, IMRT, biologically based optimization, dose-volume based optimization, plan comparison, prostate cancer, head-and-neck cancer*

***BACKGROUND***

Intensity-modulated radiotherapy (IMRT) with inverse plan optimization allows designing treatment plans with delivery of the high dose to the target volumes and steep dose gradients, while sparing critical structures. Volumetric modulated arc therapy (VMAT) promises further advantages compared to fixed-gantry IMRT techniques due to simultaneous variation of the gantry speed, dose rate and multileaf collimator (MLC) segments [1,2]. Popularity of VMAT increased dramatically after appearance and availability of the number of commercial solutions for VMAT planning and their delivery on the radiotherapy market [3-6].

Various commercial treatment planning systems feature different approaches to the VMAT optimization and sequencing, adding a certain degree of complexity in the VMAT planning process. Whereas the advantage of VMAT in terms of speed of treatment delivery is already well-known to users, the question about the quality of VMAT plans is still open and strongly depends on the approach to plan optimization and sequencing. Several planning studies were performed for the comparison of fixed-gantry IMRT vs. VMAT for different clinical sites [7-12]. Depending on the complexity of the cases and anatomical region, as well as on the applied treatment planning system, the quality of the VMAT plans ranged from inferior through equal to superior compared to IMRT plans.

However, there is evidence that application of equivalent uniform dose (EUD) formalism of the cost functions for the IMRT optimization can improve the plan quality and offer the possibility to establish class solutions for the group of cases [13-16]. Recently, efficiency of the biological formalism for VMAT was assessed by Mihaylov et.al [17]. On the other hand, these comparisons can’t provide the definitive answer about superiority of VMAT plans in terms of quality, because usually one IMRT (VMAT) plan is created per patient case. In this case there exists no guarantee that “the best possible” solution was found by the optimizer for each case.

The idea of using Pareto fronts as a tool for comparison in radiotherapy planning studies was tested by research groups of Ottosson and Petersson et al [18-20] and this evaluation concept provides a possibility to compare a set of plans minimizing such effects as planner dependence or specific plan selection. Sampling the Pareto fronts by variation of single parameter, for example maximum dose to the risk organ while keeping other parameters constant, will provide the information about optimizer efficiency in terms of fulfilling this constraint. Therefore it would be possible to evaluate and define more advantageous optimization approaches together with preferable delivery technique for a group of cases.

In the situation where resources for the treatment planning are limited (either in terms of number of workstations or time available for planning) it is important to select a planning system that provides good optimization results for the variety of clinical situations. Plan quality and delivery efficiency for VMAT together with the time required to create a desired plan become an important criteria for the choice of the TPS for VMAT implementation as a routine technique to substitute IMRT. The aim of this work was to compare different approaches to VMAT optimization (biological vs. physical DVH-based) in two commercial treatment planning systems for head-and-neck and prostate cases, using Pareto fronts. VMAT vs. IMRT Pareto front comparison was additionally performed in order to benchmark the optimizer efficiency and VMAT plan quality for each TPS. Based on the obtained results planning workflow in our institution was organized in more efficient way distributing the cases according to the anatomical group and plan intent for IMRT and VMAT cases between both systems.

***METHODS***

***Patient cases***

Three prostate and three head-and neck (H&N) cases were selected for the retrospective treatment planning study from the clinical database. CT images were acquired with a Multislice CT scanner (Siemens, Germany) in a spiral mode with 2 mm slice thickness for H&N and 4mm for prostate cases. Contouring of the cases was done in Monaco TPS to avoid differences in the systems with regard to the contour interpolation.

The cases with prostate carcinoma represented typical indication for definitive radiotherapy (low-risk, T1-T2a, Gleason score ≤6 and PSA <10). The PTV encompassed CTV (prostate gland and basis of seminal vesicles) with a margin of 1cm in all directions except posterior, where a 0.5 cm margin was specified. As organs-at-risk (OAR), bladder, rectum and femoral heads were delineated. Dose prescription was 78 Gy in 2 Gy/fx to PTV.

Three oropharynx head-and-neck cases were contoured according to the clinical protocols, respective PTVs included CTV tumor and nodal CTVs with margin of 0.5 cm. Contra-lateral parotid gland, spinal cord, brainstem and larynx were contoured as OARs. Dose prescription was 60 Gy in 2 Gy/fx to the PTV boost and 50 Gy to the nodal PTV.

Clinical plan acceptance criteria for all cases were specified as following: at least 95% of the PTV should be covered with 95% of the prescribed dose, as required by the institutional protocol, maximum dose to PTV should not exceed 115% of the prescribed dose and the doses to OARs should not exceed the values recommended by QUANTEC group reports [21-23].

***Treatment planning systems and equipment***

The study was performed using two commercially available treatment planning systems (TPS) with VMAT optimization option. First TPS, Monaco (Version 3.0.0, Elekta/CMS, USA) uses biologically constrained optimization for VMAT and IMRT, another one - Oncentra MasterPlan (V 3.3 SP3, Nucletron, NL) works only with dose-volume objectives (DVO) and constraints. In order to reduce inter-planner variability the plans in both systems were created by the same physicist with the experience in using the software in a clinical routine for 3 years. For both systems 6MV and 10MV energies from Elekta Synergy linear accelerator (Elekta Ltd, UK) with MLCi (40 leaf pairs, 1cm leaf width) were commissioned for VMAT and IMRT delivery.

***IMRT and VMAT treatment planning***

When designing treatment planning study of different planning systems, in order to provide fair comparison of the results, as many parameters as possible should be the same. We used the same beam setup, beamlet size, and a dose calculation grid, similar number of constraints in the prescription and segmentation parameters. This study design allows the assumption that observed dose differences in the planning result are mainly caused by the different optimization methods and cost function formalism in the respective systems. Additionally, application of the same parameters for the optimization and dose calculation ensures that the time needed to create a plan is approximately the same in both systems (20-30 min).

For IMRT plans 9 equidistantly spaced beams (starting from gantry angle 0°,collimator 0°) with 6MV and 10 MV energy were applied for head-and-neck and prostate cases, respectively. VMAT plans were created for the same photon energy as IMRT, using single arc with full 360° gantry rotation and collimator angle of 3°. For the purpose of the current comparison, as a starting point an IMRT plan of the same quality in terms of DVH (see *Figure 1 and 2*) was created in both systems and same prescription was used for IMRT and VMAT in both planning systems. Optimization parameters of initial plan leading to the clinically acceptable VMAT/IMRT prostate plan in Monaco and plan of the same quality in Oncentra MasterPlan (OMP) are listed in *Table 1*. *Table 2* presents the similar overview of the parameters for representative head-and-neck plan.

IMRT and VMAT plans were optimized with the beamlet width of 0.4cm. As in Oncentra MasterPlan VMAT optimization starts for gantry angle spacing of 24° [4] the same value was entered in Monaco as “Increment” in the “Beam Setup” dialog window where a user provides an input on the treatment fields (number of beams, gantry and collimator angles and selection of the treatment machine). The increment determines in how many sectors the initial full arc will be divided for the optimization process to generate fluence profiles at static gantry positions. First stage of the optimization was performed with fast pencil beam algorithm in both TPS, whereas final dose calculation was performed on 4mm^3^ grid with Monte Carlo-based (3% variance per segment/control point) and enhanced collapsed cone algorithms in Monaco and OMP, respectively. For prostate VMAT optimization and sequencing (including segment weight optimization) 91 control points were defined in both planning systems and minimal segment area was specified at 2 cm^2^ and minimal allowed number of monitor units (MU) per segment was set at 4. For prostate IMRT plans maximum 120 segments per plan were allowed in OMP, whereas in Monaco to achieve same result fluence smoothing parameter was set at 1 and „Segment suppression factor“ at value =3 to reduce number of segments to the same value approximately.

Following segmentation parameters were applied for H&N plans in both systems: 150 control points, minimal segment area = 4cm^2^, minimum 4 MU per segment. Settings limiting the number of segments in Monaco plans were set at the same values as for prostate cases.

As mentioned above, a biological formalism in terms of generalized EUD is used for the optimization functions in Monaco TPS. The main characteristic of the biological functions is that they are not point-based, in a sense that only one voxel above some threshold would violate the constraints, but rather region-based through the use of EUD, which is the dose that would have the same biological effect as the actual inhomogeneous tissue dose distribution if the tissue coverage was spatially uniform. The generalized EUD (gEUD) can be formulated as: gEUD =
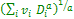
 ; where v_i_ is the fractional organ volume receiving a dose D_i_ and *a* is a tissue-specific parameter that describes the volume effect. This formula allows to consider tissue-specific property into the planning process that cannot be done with dose-volume based optimization. **Serial** cost function in Monaco TPS (used typically for the organs with serial behavior) requires to enter EUD, where value is similar to an acceptable maximum dose when the k value is large (ex. 12) and is equivalent to the mean dose when the k value is equal to 1. Power Law Exponent (k) defines a volume-effect parameter. In general, when a small k value is entered, a large volume-effect is assumed. This means that low dose volumes and high dose volumes are approximately equally weighted. When a large k value is used, there is less tolerance for excessive damage to small volumes of the assigned structure. In this case, low dose volumes receive a very small weight related to high dose volumes. **Parallel** cost function requires that user assigns three parameters. The first parameter is the Reference Dose (EUD) whose value is analogous to the dose that is only acceptable for the majority of the structures, and at which a clear dose response begins to show. The second parameter is the Mean Organ Damage to the structure in %. The Mean Organ Damage is the biological equivalent to the fraction of the volume of the structure that can be sacrificed. The third parameter is the Power Law Exponent (k). This value changes the shape of the dose response curve and determines how responsive the structure is to the Reference Dose and Mean Organ Damage values entered. A higher k value translates to a steep dose response that often translates into a pronounced kink in the DVH curve [13-16]. Readers are referred to vendor-provided manuals or training for more detailed descriptions about those cost functions.

***Plan evaluation and construction of Pareto fronts***

Evaluation of the plans was performed according to the recent ICRU Report 83 recommendations [24]; additionally relevant dose-volume parameters from QUANTEC reports were assessed. For PTVs minimum, maximum, and median doses were reported. As surrogates for minimum and maximum dose, the dose to 98% and 2% of the target volume were evaluated. In addition, we reported tumor coverage as percentage of the respective PTV volume covered with 95% of the prescribed dose for all VMAT and IMRT plans. The target conformity index (CI), as proposed by Paddick [25], was evaluated for the PTV volume covered with 95% isodose.

$CI=\frac{(V_{PTV}^{95\%D})^{2}}{V_{PTV}\cdot V_{95\%D}}$,

where V_PTV_ stands for the PTV volume in ccm, V_95%D_ refers to the total volume covered with 95% isodose line and V_PTV_^95%D^ corresponds to the part of the PTV volume covered with 95% isodose.

In CI, target homogeneity index (HI), defined as

$HI=\frac{D_{2\%}-D_{98\%}}{D_{median}}$, was calculated.

For OARs maximum dose (as well taken as dose to 2% of the OAR volume), median or mean doses were used for comparison. In prostate cases rectum volume covered by 60Gy (V_60Gy_) and for bladder V_65Gy_ were assessed. In H&N cases following OAR dose-volume indices were evaluated: D_2%_ for spinal cord and brainstem, and mean dose for contra-lateral parotid gland. In all VMAT and IMRT plans, volume of the patient covered with 5Gy was recorded.

Again in order to reduce the error caused by different methods of DVH calculation in both TPS, calculated dose was imported in Monaco TPS and DVH curves and parameters were calculated with 1 mm resolution and 0.1 Gy dose bins. Number of segments (control points) and monitor units (MU) was reported for each plan as well.

A treatment plan is Pareto optimal for a given set of objectives and constraints if there is no other feasible treatment plan that is at least as good in all the objectives and strictly better in at least one objective. A Pareto front is constituted by the Pareto optimal solutions and comparison of the sampled Pareto fronts, rather than individual treatment plans, gives better picture about abilities of the TPS to achieve required planning objectives.

Pareto fronts were created by adjusting the optimization constraints of the initial clinical acceptable plan (as shown in *Tables 1* and *2*) for one of the organs-at-risk of, while keeping other parameters constant. As Monaco employs the biological EUD-based formalism of the cost functions for OAR optimizations, we have chosen to vary EUD value in Serial or “Mean Organ damage %” value in Parallel cost function, whereas in OMP one DVO value was changed. For each case 15 plans were optimized for the front sampling, until the plan acceptance criteria in terms of PTV coverage were violated.

In prostate cases for rectum we consecutively lowered Serial EUD value or DVO “Max dose to 20% of the volume” by 0.5 Gy in Monaco and OMP, respectively. Pareto fronts were sampled based on the clinical acceptance criteria for the PTV coverage and relative rectum volume receiving 60Gy. Assuming that biologically constrained cost function works on the whole DVH curve opposite to the DVO optimization concept, median dose to rectum was evaluated as well. In head-and-neck cases we changed for parotid gland volume percentage “Mean Organ damage %” value in Parallel cost function in Monaco in 3 % steps starting from the parameters in Table 2. In OMP DVO for percentage of volume receiving 25 Gy was adjusted. Pareto fronts were constructed for the mean dose to parotid gland vs. PTV coverage by 95% isodose.

***RESULTS***

*Prostate*

Variation of the dose-volume constraint for rectum, together with PTV coverage is reported in *Table 3*. Pareto fronts, based on these parameters for all three prostate cases, are shown in the *Figure 3*. In case of OMP, quality of IMRT plans in terms of rectum sparing and PTV coverage was superior to VMAT solutions for all three cases. For Monaco, this trend was confirmed for Pro-1 case, whereas for Pro-2 and Pro-3 cases VMAT and IMRT plans resulted in very similar plan quality.

In the intersystem comparison for IMRT plans, for the cases with significant dose reduction to rectum and still above PTV coverage over 95% both systems generated very similar solutions. With regard to VMAT plans, optimization solutions from Monaco were better for rectum sparing and PTV coverage than OMP, as shown by solid black trend lines for all three cases.

*Table 4* shows the variation for the dose-volume indices observed while creating Pareto fronts for prostate cases. Front sampling process did not influence much median and maximum PTV doses, as shown by small standard deviations in the *Table 3*. The influence on the plan conformity and homogeneity was minimal as well. Both TPS demonstrated very similar homogeneity in VMAT and IMRT plans, except OMP VMAT plans for Pro-2 case. This can be explained by large intersection between PTV and rectum volumes. Plan conformity indices were similar for IMRT and VMAT plans as well, but conformity of the Monaco solutions was much better (10% and more) compared to OMP.

For the rectum and bladder, choice of the delivery technique and TPS had almost no influence for D2% in rectum and bladder. With regard to the median dose to rectum, in both systems VMAT plans resulted in 2-5% lower values compared to IMRT. However, while evaluating Dmedian and V65Gy of the bladder, very large difference was demonstrated for EUD-based optimization compared to OMP. Median doses to bladder were 10 to 20% lower in case of Monaco in VMAT and IMRT plans (see *Table 4*). Volume of the non target tissue receiving 5Gy and more was quite different between both delivery techniques and planning systems. In case of Monaco, VMAT plans resulted in 300-500 ccm larger V5Gy. For OMP only in Pro-1 case VMAT plans resulted in larger 5Gy volume, for two other cases VMAT plans resulted in over 800 ccm lower V5Gy. In the intersystem analysis, almost no difference in 5Gy volume was demonstrated in VMAT plans, whereas Monaco IMRT plans showed lowest values in all cases.

The variation of the parameters influencing the delivery efficiency is presented in the *Table 5*. The number of segments in Monaco IMRT plans, as well as number of MUs, was constantly higher compared to OMP. Due to specifics of VMAT sequencer in Monaco with variable number of control points per arc, solutions with either more or less controls points, compared to OMP are presented and similar to IMRT cases show more MUs per arc. The difference in MU between VMAT and IMRT plans was below 100 MU in both systems for all three cases.

**Head-and-neck**

Variation of the mean dose constraint to parotid gland, together with PTV coverage is reported in *Table 3*. Pareto fronts, based on these parameters for all three head-and-neck cases, are shown in the *Figure 4*. In both systems, IMRT solutions were superior to VMAT in regions with excessive parotid sparing. Intersystem comparison showed very similar IMRT plan quality for HN1 and HN2 cases, in third case OMP solutions were better than Monaco.

*Table 6* shows the variation for the dose-volume indices observed while creating Pareto fronts for head-and-neck cases. Similar to the prostate cases Pareto front sampling process did not influence much median and maximum PTV doses, however coverage of the PTV boost, was sometimes compromised. D2% for spinal cord and brainstem were either better or similar for IMRT plans in both systems. Volume of normal tissue, receiving 5Gy or more was very similar between both systems and delivery techniques, again with lowest values in Monaco IMRT plans. Solutions obtained with Monaco IMRT planning resulted in best target homogeneity, as shown in *Table 6*. Plan conformity was quite similar in both TPS with variations around 5% between different delivery techniques.

Delivery efficiency of IMRT plans in terms of segments and monitor units as shown in *Table 5*, was slightly better for Monaco compared to OMP. Difference between VMAT and IMRT MUs was again very small in case of Monaco, whereas for OMP approximately 250 MU less was needed for VMAT plans.

***DISCUSSION***

Pareto fronts represent valid and convenient tool to explore potential of the optimizer in different planning systems to achieve desirable balance between doses to target and OARs. From clinical point of view, data obtained with Pareto fronts can provide an estimate of the potential reduction of OAR dose and therefore reduce complication probability. The advantage of using biological models in treatment planning is that all the voxels present in the region of interest are playing a role in the optimization process instead of the few outlier voxels that receive a disproportionate optimization weight due to point-based constraint violations. Biological cost functions offer more control over the dose distribution than physical cost functions, and a smaller number of them is required to fully shape the dose distribution.

The question, which treatment technique (IMRT or VMAT) is preferable in terms of target coverage and organ sparing was discussed controversially in the literature [7-12, 26]. Comparison with the published data was difficult, because these studies employ different treatment planning systems and do not use Pareto fronts as plan analysis tool. In case of Oncentra MasterPlan, published data suggest better target coverage and OAR sparing for single arc VMAT for prostate cancer compared to IMRT, whereas for head-and-neck use of dual-arc was recommended to achieve results comparable with IMRT [4, 27].

In the situation where resources are very limited (number of workstations for planning, large patient throughput) a choice of the TPS is important to avoid delays in the clinical routine during planning process and also to be able to provide advantageous planning solutions for the given clinical case.

As can be seen in our study Pareto fronts for prostate cases show that single-arc VMAT plans are worse compared to 9 field IMRT plans for Oncentra Master Plan, whereas for Monaco TPS this difference in plan quality was minimal. With regard to other parameters used for plan evaluation, in case of prostate plans, homogeneity and conformity indices together with median bladder dose and lower 5Gy volume indicate clinically better solutions created with Monaco, however this benefit is slightly compromised by the reduced delivery efficiency of IMRT plans. Taking all factors and delivery efficiency into account, VMAT delivery based on Monaco-optimized prostate plans was selected as preferable option in our institution.

For head-and neck case NH2 where very strong modulation was required due to proximity of the OARs and large overlap of the parotid gland with PTV, VMAT plans done with Oncentra were inferior compared to IMRT solutions. This confirms that for cases where strong modulation is required dual-arc VMAT or IMRT would be preferable option. In two other cases IMRT and VMAT solutions of OMP were quite similar. In Monaco system both IMRT and VMAT plans were showing same plan quality, with results close to IMRT plans done with Oncentra except HN3 case. In our opinion, here good solution could be possibly achieved with Monaco TPS if we would allow more control points per arc and consequently more MUs to achieve similar modulation. In general, for all head-and-neck cases, Monaco provided better target dose homogeneity and conformity and only small increase of the dose to spinal cord and brainstem within clinically acceptable range. Based on these findings we prefer use of IMRT in Oncentra or Monaco TPS when strong modulation is required in complex head-and-neck cases and VMAT in less complex situations where planning aims can be achieved with single-arc VMAT. Due to fast planning process (DVH control by biological cost functions) and ability to create class solutions (plan templates) with Monaco system VMAT planning is less time consuming. With regard to the efficiency of plan delivery more conformal dose distributions obtained with Monaco are achieved by the system due to the higher degree of modulation in the plans and consequently require more MUs.

In our opinion possible expected gain in doses to OARs in highly complex plans with use of biological cost-functions in Monaco was compromised by VMAT sequencer in this version of TPS as also noticed in the paper of Nevelsky at al [28]. Hence speed of the treatment delivery is reduced with VMAT by approximately 60% [2,4,5,10], the data obtained in this study, shows that single-arc VMAT represents valid alternative to static 9-beams IMRT in both prostate and head-and-neck cases.

As was pointed out it is difficult to design a treatment planning study for systems due to the differences in the implementation of optimization and sequencing algorithms. Comparison between optimization results done before sequencing would provide better insight on the advantages of the biological cost functions, however, without a creation of deliverable plans these results would have little practical value. Sequencing for VMAT in Monaco and Oncentra and inability to change flexibly number of control points per arc in OMP can be considered as a major limitation of the system. On the other hand absence of segment shape optimization in the version 3.00 of Monaco produced large difference between fluence obtained in the first stage of the optimization and final result. This feature was implemented in later releases and expected to improve further MU efficiency of the Monaco VMAT plans.

***CONCLUSIONS***

It was found in this study that for the prostate cases VMAT plans generated with Monaco TPS showed the same dosimetric quality as 9 beam IMRT plans. In the Oncentra system the difference was much more pronounced in favor of IMRT solutions. Compared with Oncentra system Monaco VMAT plans also reduced median dose to bladder and showed a better target conformity due to the use of biological cost functions. For head-and-neck cases with complex anatomy where strong modulation is desired, static beam IMRT plans or VMAT plans with large number of control points per arc (120 or above) and dual arc VMAT technique would be preferred in both treatment planning systems. Limitations of the sequencer reduced the potential advantage of the biological optimization for head-and-neck plans and produced similar results in intersystem comparison.

***COMPETING INTERESTS***

Redacted

***REFERENCES:***

1. Yu CX, Tang G: Intensity-modulated arc therapy: principles, technologies and clinical implementation. Phys Med Biol 2011, 56: R31–R54
2. Bedford JL: Treatment planning for volumetric modulated arc therapy. Med Phys 2009, 36(11):5128-5138.
3. Rao M, Yang W, Chen F, et al: Comparison of Elekta VMAT with helical tomotherapy and fixed field IMRT: plan quality, delivery efficiency and accuracy. Radiation Oncology 2010, 5:110
4. Alvarez-Moret J, Pohl F, Koelbl O, et al: Evaluation of volumetric modulated arc therapy (VMAT) with Oncentra MasterPlan® for the treatment of head and neck cancer. Radiat Oncology 2010, 5:110
5. Bertelsen A, Hansen CR, Johansen J, et al Single Arc Volumetric Modulated Arc Therapy of head and neck cancer. Radiother Oncol 2010, 95: 142–148
6. Pesce G, Clivio A, Cozzi L, et al: Early clinical experience of radiotherapy of prostate cancer with volumetric modulated arc therapy. Radiation Oncology 2010, 5:54
7. Clemente S, Wu BB, Sanguineti G, et al: SmartArc-based volumetric modulated arc therapy for oropharyngeal cancer: a dosimetric comparison with both intensity-modulated radiation therapy and helical tomotherapy. Int J Rad Oncol Biol. Phys 2011, 80: 1248–1255
8. Tang G, Phil M, Earl MA, et al: Comparing radiation treatments using intensity-modulated beams, multiple arcs, and single arcs. Int J Rad Oncol Biol. Phys. 2010, 76:1554–1562.
9. Davidson MT, Blake SJ, Batchelar DL, et al: Assessing the role of volumetric modulated arc therapy (VMAT) relative to IMRT and helical tomotherapy in the management of localized, locallyadvanced, and post- operative prostate cancer. Int J Rad Oncol Biol Phys 2011:1–9
10. Wiezorek T, Brachwitz T, Georg D, et al: Rotational IMRT techniques compared to fixed gantry IMRT and Tomotherapy: multi-institutional planning study for head-and-neck cases. Radiation Oncology 2011, 6:20
11. Wolff D, Stieler F, Welzel G, et al: Volumetric modulated arc therapy (VMAT) vs. serial tomotherapy, step-and-shoot IMRT and 3D-conformal RT for treatment of prostate cancer. Radiother Oncol 2009, 93:226–233
12. Palma D, Vollans E, James K, et al: Volumetric modulated arc therapy for delivery of prostate radiotherapy: complication with intensity-modulated radiotherapy and three-dimensional conformal radiotherapy Int J Rad Oncol Biol. Phys 2008, 72: 996–1001
13. M. Alber and F. Nuesslin: An objective function for radiation treatment optimization based on local biological measures. Phys Med Biol, 44:479-493, 1999.
14. Thieke C, Bortfeld T, Niemierko A, et al: From physical dose constraints to equivalent uniform dose constraints in inverse radiotherapy planning. Med Phys 2003, 30 (9): 2332-2339
15. Semenenko VA, Reitz B, Day E, et al: Evaluation of a commercial biologically based IMRT treatment planning system. Med Phys 2008, 35 (12):5851-5860
16. Qi XS, Semenenko VA, Li XA: Improved critical structure sparing with biologically based IMRT optimization. Med Phys 2009, 36(5): 1790–1799
17. Mihaylov IB, Fatyga M, Bzdusek K, et al: Biological optimization volumetric modulated arc radiotherapy for prostate carcinoma. Int J Rad Oncol Biol. Phys 2010
18. Ottosson RO, Engstrom PE, Sjostrom D, et al: The feasibility of using Pareto fronts for comparison of treatment planning systems and delivery techniques. Acta Oncol 2009, 48: 233-237
19. Ottosson RO, Karlsson A, Behrens CF: Pareto front analysis of 6 and 15 MV dynamic IMRT for lung cancer using pencil beam, AAA and Monte Carlo. Phys Med Biol 2010, 55: 4521–4533
20. Petersson K, Ceberg C, Benedek H, et al: Conversion of helical tomotherapy plans to step-and-shoot IMRT plans—Pareto front evaluation of plans from a new treatment planning system. Med Phys 2011, 38 (6): 3130-3138
21. Quantitative Analyses of Normal Tissue Effects in the Clinic. (Supplement 1). Int J Radiat Oncol Biol Phys 2010, 76(3):1-160
22. Viswanathan AN, Yorke ED, Marks LB et al: Radiation dose-volume effects of the urinary bladder. Int J Radiat Oncol Biol Phys 2010, 76(3):1116-122,.
23. Michalski J, Gay H, Jackson A, et al: Radiation dose-volume effects in radiation-induced rectal injury. Int J Radiat Oncol Biol Phys 2010, 76(3):123-129
24. ICRU-Report 83. Prescribing, Recording, and Reporting Photon-Beam Intensity-Modulated Radiation Therapy (IMRT). Journal of the ICRU, 10(1), 2010.
25. Paddick I: A simple scoring ratio to index the conformity of radiosurgical treatment plans. J Neurosurg, 93 Suppl 3:219-222, 2000.
26. Doornaert P, Verbakel WF, Bieker M et al: RapidArc Planning and Delivery in Patients with Locally Advanced head-and-neck Cancer Undergoing Chemoradiotherapy. Int J Radiat Oncol Biol Phys 2011, 79(2):429-435.
27. Dobler B, Weidner K, Koelbl O: Application of volumetric modulated arc therapy (VMAT) in a dual-vendor environment. Radiation Oncology 2010, 5:95
28. Nevelsky, A., Ieumwananonthachai, N., Kaidar-Person, O., et al: Hippocampal-sparing whole-brain radiotherapy using Elekta equipment. Journal Of Appl Clin Med Phys, 14(3). doi:10.1120/jacmp.v14i3.4205

***FIGURE LEGENDS***

Figure 1. Representative dose distributions for prostate VMAT and IMRT plans in both treatment planning systems.

Figure 2. Representative dose distributions for prostate VMAT and IMRT plans in both treatment planning systems.

Figure 3. Pareto fronts for prostate cases. Symbols with dotted contour and light grey filling indicate non-Pareto optimal solutions, provided by optimizer.

Figure 4. Pareto fronts for head-and-neck cases. Symbols with dotted contour and light grey filling indicate non-Pareto optimal solutions, provided by optimizer.

***TABLES***

Table 1. Summary of optimization prescription for prostate VMAT and IMRT cases in Monaco and Oncentra MasterPlan treatment planning systems.

Dose optimization to OARs in Monaco was performed only with biological cost functions, optimization in OMP was done with dose-volume objectives. EUD=equivalent uniform dose, k=parameter for penalty strength, regulating steepness of the DVH curve

Table 2. Summary of optimization prescription for head-and-neck VMAT and IMRT cases in Monaco and Oncentra MasterPlan treatment planning systems.

Dose optimization to OARs in Monaco was performed only with biological cost functions, optimization in OMP was done with dose-volume objectives. EUD=equivalent uniform dose, k=parameter for penalty strength, regulating steepness of the DVH curve.

Table 3. Data for generation of Pareto fronts. Range of PTV coverage, rectal volume constraints and mean parotid gland doses, for prostate and head-and-neck cases respectively.

Table 4. Overview of the dose-volume parameters for prostate plans. Data presented as mean± standard deviation for all created plans.

Table 5. Delivery efficiency of prostate & head-and-neck plans. Number of segments (control points) is reported as range, for MUs additionally mean± standard deviation is

reported.

Table 6. Overview of the dose-volume parameters for head-and-neck plans. Data presented as mean± standard deviation for all created plans.
